# The Effect of How Outcomes Are Framed on Decisions about Whether to Take Antihypertensive Medication: A Randomized Trial

**DOI:** 10.1371/journal.pone.0009469

**Published:** 2010-03-01

**Authors:** Cheryl L. L. Carling, Doris Tove Kristoffersen, Andrew D. Oxman, Signe Flottorp, Atle Fretheim, Holger J. Schünemann, Elie A. Akl, Jeph Herrin, Thomas D. MacKenzie, Victor M. Montori

**Affiliations:** 1 Norwegian Knowledge Centre for the Health Services, Oslo, Norway; 2 Clinical Research and INFORMAtion Translation Unit, and Department of Epidemiology, Italian National Cancer Institute Regina Elena, Rome, Italy; 3 Department of Medicine, University at Buffalo, Buffalo, New York, United States of America; 4 Division of Cardiology, Yale University, New Haven, Connecticut, United States of America; 5 Department of Internal Medicine, Denver Health and Hospital Authority and University of Colorado Health Sciences Center, Denver, Colorado, United States of America; 6 Knowledge and Encounter Research Unit, Division of Endocrinology and Internal Medicine, Mayo Clinic College of Medicine, Rochester, Minnesota, United States of America; Cardiff University, United Kingdom

## Abstract

**Background:**

We conducted an Internet-based randomized trial comparing three valence framing presentations of the benefits of antihypertensive medication in preventing cardiovascular disease (CVD) for people with newly diagnosed hypertension to determine which framing presentation resulted in choices most consistent with participants' values.

**Methods and Findings:**

In this second in a series of televised trials in cooperation with the Norwegian Broadcasting Company, adult volunteers rated the relative importance of the consequences of taking antihypertensive medication using visual analogue scales (VAS). Participants viewed information (or no information) to which they were randomized and decided whether or not to take medication. We compared positive framing over 10 years (the number escaping CVD per 1000); negative framing over 10 years (the number that will have CVD) and negative framing per year over 10 years of the effects of antihypertensive medication on the 10-year risk for CVD for a 40 year-old man with newly diagnosed hypertension without other risk factors. Finally, all participants were shown all presentations and detailed patient information about hypertension and were asked to decide again. We calculated a relative importance score (RIS) by subtracting the VAS-scores for the undesirable consequences of antihypertensive medication from the VAS-score for the benefit of CVD risk reduction. We used logistic regression to determine the association between participants' RIS and their choice. 1,528 participants completed the study. The statistically significant differences between the groups in the likelihood of choosing to take antihypertensive medication in relation to different values (RIS) increased as the RIS increased. Positively framed information lead to decisions most consistent with those made by everyone for the second, more fully informed decision. There was a statistically significant decrease in deciding to take antihypertensives on the second decision, both within groups and overall.

**Conclusions:**

For decisions about taking antihypertensive medication for people with a relatively low baseline risk of CVD (70 per 1000 over 10 years), both positive and negative framing resulted in significantly more people deciding to take medication compared to what participants decided after being shown all three of the presentations.

**Trial Registration:**

International Standard Randomised Controlled Trial Number Register ISRCTN 33771631

## Introduction

How information about treatment effects is presented affects how it is understood and subsequent decisions [1]–[3]. When decisions are preference sensitive [4], i.e. where individual preferences about the desirable and undesirable consequences determine choice, it is important to provide patients with information in a format that facilitates decisions that are consistent with their values and preferences [5]–[9].The aim of the Health Information Project: Presentation Online (HIPPO) was to improve communication of information about the effects of health care based on randomized trials of alternative ways of presenting this evidence, in order to determine which presentations help people make decisions that are consistent with their values.

“Decision frame” refers to the decision-maker's conceptualization of the decision problem and all its attributes, e.g. outcomes and contingencies. This is partly dependent on the decision-maker's personal characteristics and partly on the way the problem is formulated. Framing studies can manipulate logically equivalent information or give more or less the same information though not logically equivalent [3], [10]. Information about health effects can be framed either in terms of potential gains (advantages or benefits), called positive framing, or in terms of potential losses (disadvantages or harms), called negative framing. The “valence framing effect” occurs when individuals' choices vary depending on whether outcome information is presented in a negative or a positive light [11], [12], as opposed to “pure framing”, which occurs when outcomes and their probabilities are presented with different wordings or formats that are objectively equivalent. [13]. The framing postulate of prospect theory suggests that people respond differentially to messages depending on how these messages are framed. Although the information presented is equivalent, the willingness to incur risk in order to promote a desirable outcome or avoid an undesirable outcome differs [14], [15].

Hypertension is associated with increased risk for events that are manifestations of serious cardiovascular disease (CVD), including myocardial infarction and stroke [16]. The focus of this trial was on how framing of the benefits of taking antihypertensive medication for people with hypertension affects their decisions about whether or not to start taking medication. We chose this decision because it is a common problem of broad interest and many patients prefer not to take treatment for mild hypertension if the advantages and disadvantages are explained [17]. The objective was to compare the impact of how the information was framed on decisions about whether to take medication in relation to the values of the participants. Values here refers to the relative desirability of the possible consequences of a healthcare intervention, including health outcomes (such as CVD and the side effects of antihypertensive medication), the burden of treatment (such as the inconvenience of taking antihypertensive medication daily), and resource expenditures [18].

The main benefit of reducing high blood pressure is the reduction of risk for serious cardio-vascular events such as stroke and myocardial infarction [19], a preventive behaviour. According to prospect theory [14], choosing a preventive behaviour would be described as a risk-averse option, which people prefer when gains are made salient. Therefore, one would expect a larger proportion of people to choose to take antihypertensive medication if information was positively rather than negatively framed. However, empirical evidence does not consistently support this hypothesis [2].

We are not aware of any previous studies that have compared the effects of positive and negative framing on the extent to which people's decisions are consistent with their values. Thus, we designed this study to assess the extent to which positive and negative framing affect choices about whether to take medication for hypertension. Although it has been shown that how information is presented can influence patients' decisions, it is not clear how best to inform patients in this situation [17]. Although natural frequencies may be better understood and preferred [20], natural frequencies can be presented either positively (the frequency of CVD not occurring) or negatively (the frequency of CVD occurring). Frequencies can also be presented over different timeframes (the frequency of CVD per year or the frequency of CVD for 10 years). There is high quality evidence of the effects of antihypertensive medication on CVD [19], but not for how to present this evidence to patients. Because it is a preference sensitive decision that is affected by patients' values, one would expect some degree of correlation between how important the desirable and undesirable consequences of taking antihypertensive medication are to them and the likelihood that they would decide to take medication. In other words, one would expect that people for whom the benefits of taking antihypertensive medication were more important and the downsides less important would be more likely, on average, to decide to take medication than people for whom the benefits were less important and the downsides were more important.

## Methods

### Ethics Statement

This study was approved by the Norwegian Data Protection Agency, the Norwegian Medical Ethics Board and the Health Sciences Institutional Review Board (HSIRB) of the University at Buffalo.

The CONSORT checklist and the protocol for this study are available as supporting information; see Checklist S1 and Protocol S1.

**Figure 1:**
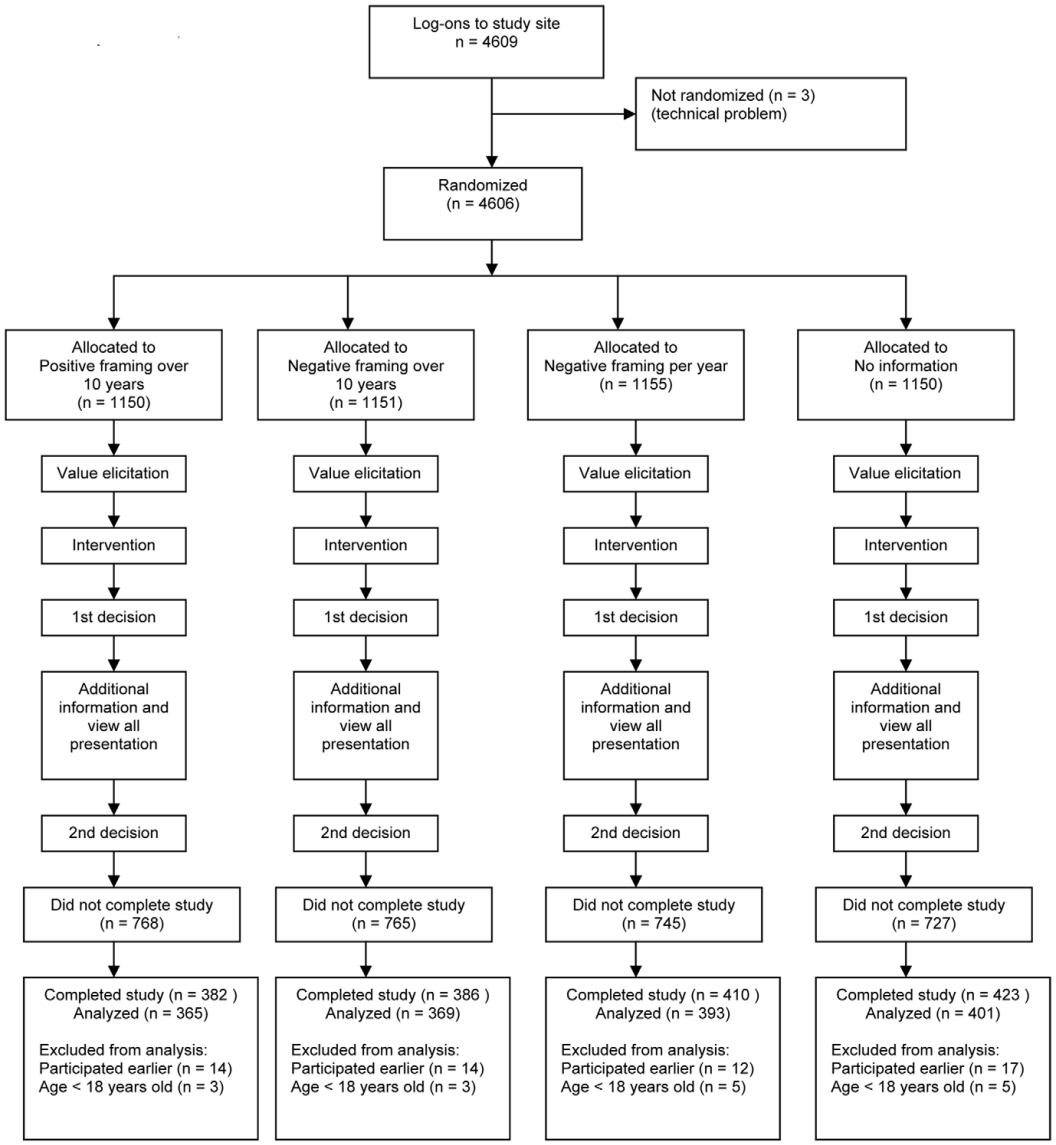
CONSORT flow-chart.

The study was an Internet-based randomized trial in which participants were randomized to one of three ways of framing information about the effects of antihypertensive medication on the 10-year risk of cardiovascular disease (CVD) or to no information (Figure 1 Consort flow-diagram). The estimate of CVD risk without antihypertensive medication was based on Framingham data [21] for a 40 year-old man with blood pressure of 160/95 without other risk factors. Because a 40 year-old man would have a low risk for stroke, we estimated the benefit over 10 years based on a 20% relative risk reduction in CVD [19]. We selected this scenario because it was within the lower range of risk levels for which antihypertensive medication is commonly recommended [22] and we assumed that decisions whether to take antihypertensive medication are more preference sensitive when the risk of CVD is relatively low.

### Interventions and Comparisons

**Figure 2:**
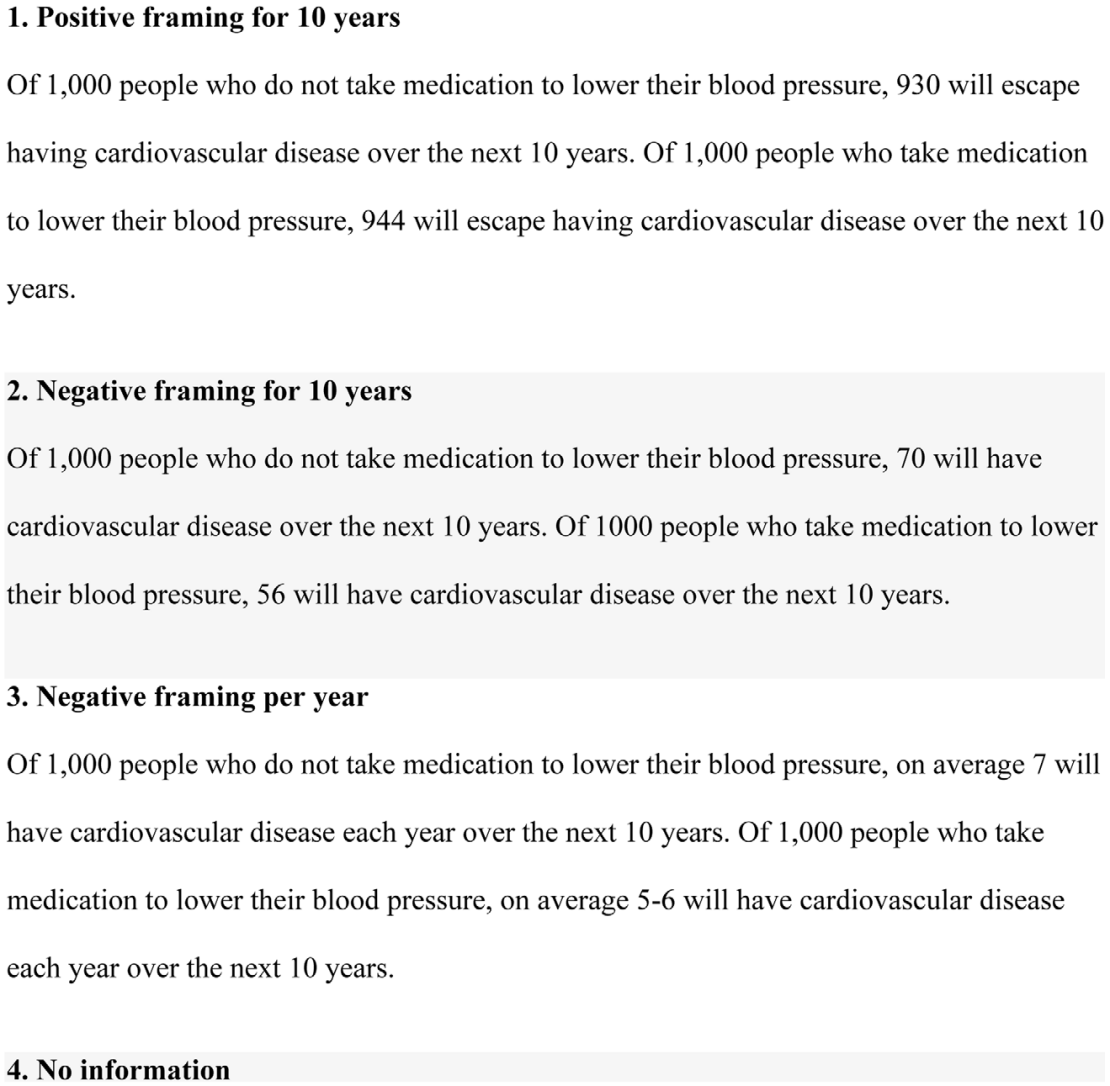
Presentations of benefits of taking medication for hypertension.

We evaluated the following three ways of framing the information: 1. positively framed information showing gain over 10 years (positive framing for 10 years); 2. negatively framed information showing loss over 10 years (negative framing for 10 years), and 3. negatively framed information showing loss per year over 10 years (negative framing per year) (Figure 2). We included the third group to determine whether a shorter time frame with correspondingly fewer events would affect participants' decisions.

We planned two main comparisons in advance: 1. positively versus negatively framed information over 10 years, and 2. negatively framed information per year versus negatively framed information over 10 years.

### Study Design

Information about the study was broadcast on Puls, a popular nationally televised weekly health program with approximately 700,000 viewers (total population of Norway = 4.5 million). On the program, we presented documentation regarding the use of antihypertensive medication in Norway and invited viewers to go to our website to participate in the study. A reminder was broadcast on the program after a few weeks.

The website was in Norwegian. Upon logging on participants were presented with information about the study and asked to give informed consent by clicking on an arrow in order to proceed and participate in the study. The participants viewed a brief scenario in which each was asked to imagine that he or she was a 40 year-old man who does not smoke, is active and has a healthy diet. The doctor tells him that he has high blood pressure and therefore has an increased risk of cardiovascular disease, particularly stroke and heart attack. Explanations were available for terms such as high blood pressure and stroke using hypertext links.

**Figure 3:**
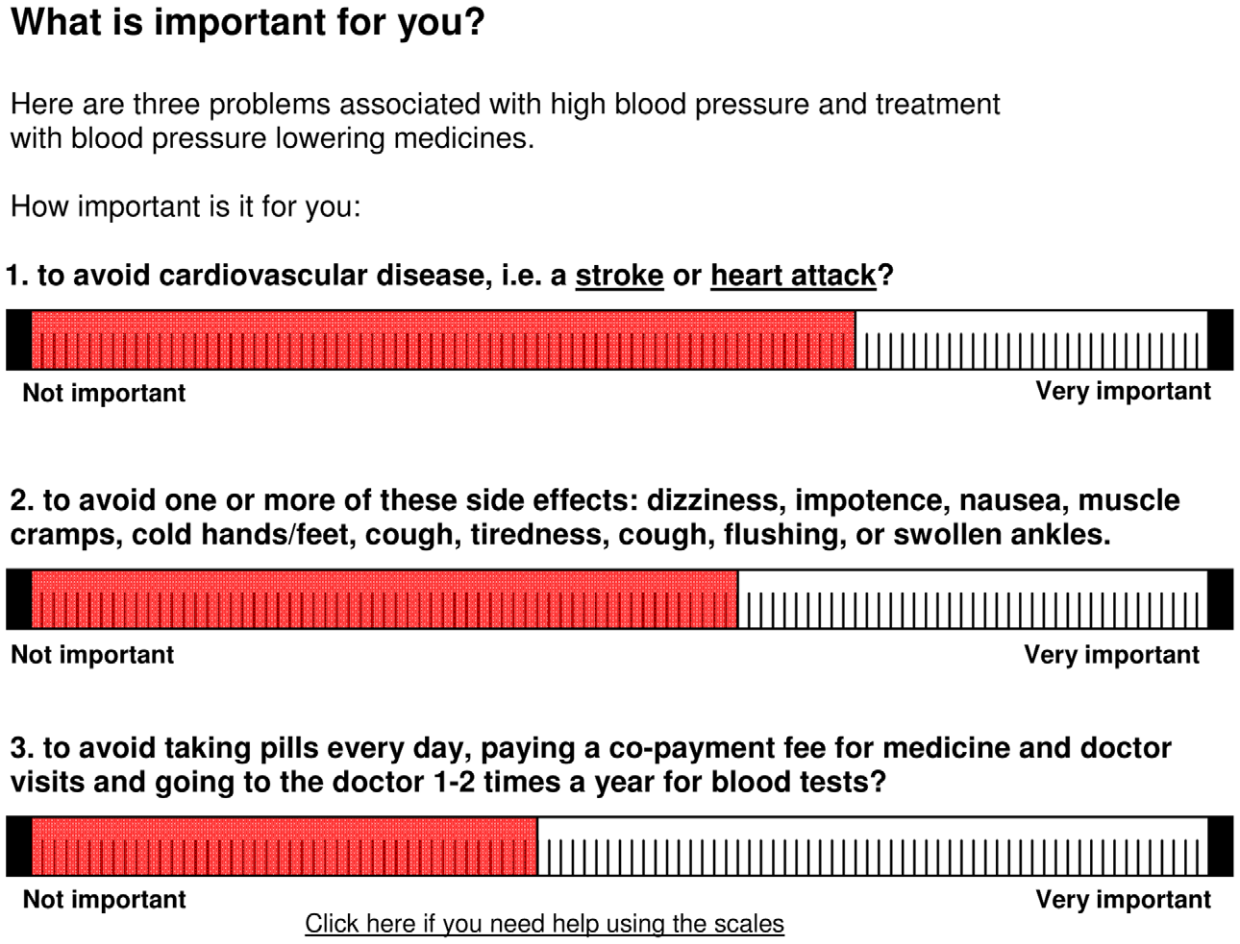
Visual analogue scales (VAS) used to elicit participants' preferences. Translation of value elicitation instrument, which was presented in Norwegian. Pop-up descriptions of stroke and heart attack were provided if participants clicked on the hypertext links.

We then asked participants to indicate the relative importance of three consequences of hypertension and its treatment: avoiding CVD (stroke and heart attack), the side effects of antihypertensive medication (which were listed), and the inconvenience associated with taking antihypertensive medication (taking pills every day, co-payments for the medication, and going to the doctor 1–2 times per year) using horizontal 100-point visual analogue scales (VAS) (Figure 3). The lower and upper anchors of the VAS were labelled “Not important” and “Very important”.

**Figure 4:**
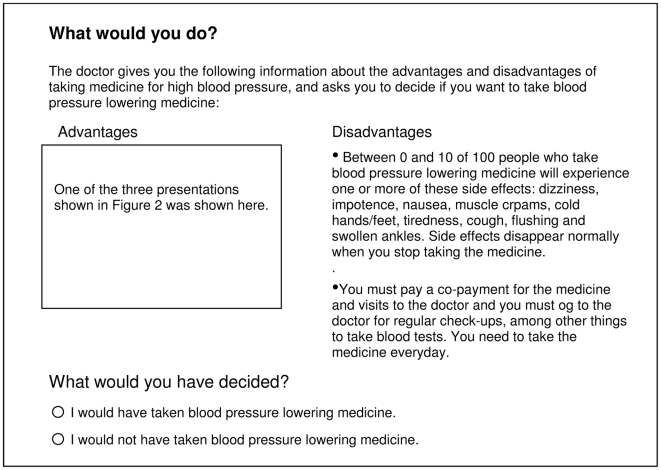
Presentation of advantages and disadvantages of antihypertensive medication and decision elicitation. Translation of the information, which was presented in Norwegian. Participants randomised to “no information” were not shown any information about the advantages or disadvantages of taking antihypertensive medication.

**Figure 5:**
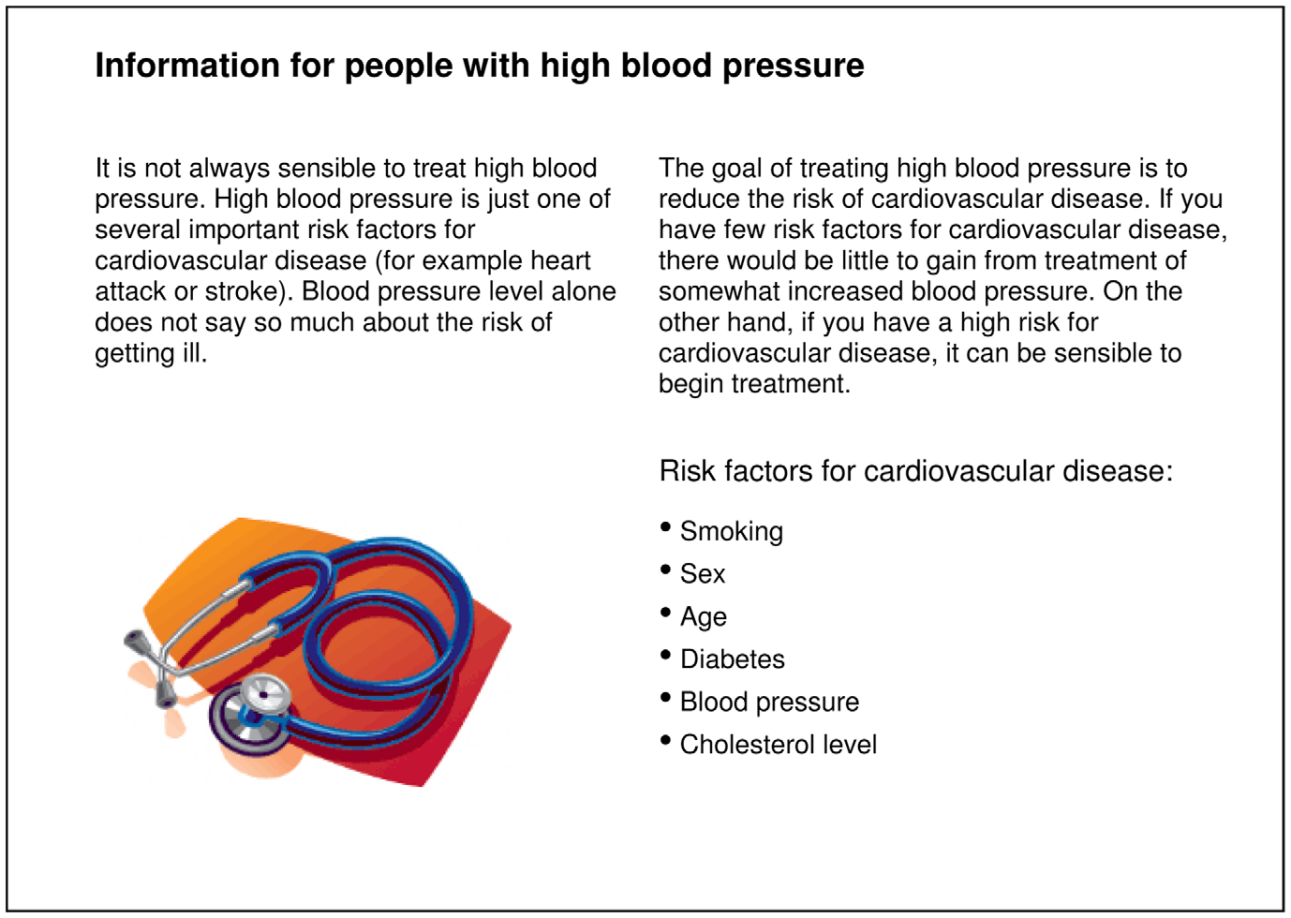
Information presented to participants prior to their second decision. Translation of the information, which was presented in Norwegian.

Participants then viewed one of the three presentations of the advantages of antihypertensive medication and a standard presentation of the disadvantages or received no information (Figure 4), based on random allocation. When the participants logged-on to the study, the system randomised them, using block randomisation with a sequence of 100 blocks of four that was generated on http://www.randomization.com. After viewing the presentation to which they were allocated or receiving no information, participants were asked to indicate whether they would or would not take antihypertensive medication with two response options: yes or no (Figure 4). We then asked their sex, age and years of education using drop-down response options. Afterwards, all participants were shown additional information about hypertension and its treatment (Figure 5), shown all three presentations in a block-randomized sequence, and asked to reconsider their original decision and indicate anew if they would take antihypertensive medication.

Responses from participants who stated that they were at least 18 years-old and that they were filling in the questionnaire for the first time were included in the analysis. Participants' responses to the questions on our website were directly saved into a database where the data were stored anonymously. Confidentiality of data was ensured by not collecting any information that would make it possible to identify the participants. Voluntary contact information that some participants supplied in order to be informed of future studies was stored in a separate database; thus it was not possible to couple contact information and study data. Participants were informed on the consent screen that they could leave the study at any time, and they were given the option of choosing to have any data that they might have entered deleted.

### Analysis and Sample Size

For each participant, we calculated a Relative Importance Score (RIS), by subtracting the sum of her VAS-scores for the relative importance of avoiding the downsides of antihypertensive medication (side effects and inconvenience) from her VAS-score for the relative importance of avoiding cardiovascular disease. We expected that higher RIS would be correlated with an increased likelihood of deciding to take medication.

We used logistic regression to compare the effects of the different presentations on the decision to take medication, with the decision to take medication (yes or no) as the dependent variable, and the RIS and allocated presentation as predictors. The following model was used:
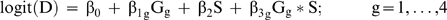
where D is the decision to take medication or not, G is the presentation group, S is the RIS value and G*S is the interaction between the presentation and the RIS value. To make inferences about the response within each group and for the comparisons of groups we used dummy variable coding with reference parameterization for the presentation groups, i.e. directly estimating the difference in the effect between the presentation group and the reference group, i.e. negative framing for 10 years. Wald tests were used for the *p*-values and confidence intervals from the logistic regression and chi-square tests were used to compare frequencies.

Based on the results of previous studies [20], [23], [24], we estimated we would need about 350 participants per group to achieve 80% power at a significance level of 0.025 after applying a Bonferroni correction for the two main comparisons (α = 0.05/2); i.e. for the comparison of the slope of the linear predictors for the group of positively versus the group of negatively framed information over 10 years and for the group of negatively framed information per year versus the group of negatively framed information over 10 years. It cannot be assumed that the presentation group with the steepest slope resulted in choices that were most consistent with participants' values. We therefore also planned comparisons of the difference in log odds at 1st and 3rd quartiles and the median values of RIS.

We also considered which group made decisions that were the most consistent with the “more fully informed” second decision, made by the participants after they had seen all three presentations and been provided more detailed information. This was done by comparing the linear predictor for each group for the first decision with the linear predictor (pooled estimate) across the other three groups for the second decision, using the model above without the interaction term. We used a logistic regression model to explore whether the respondents changed their decision from ‘Taking medication’ to ‘Not taking medication’ versus ‘Did not change decision’ depending on the RIS, presentation group, and their interaction.

## Results

There were 4,609 log-ons to the study website between November 2004 and May 2005 (Figure 1). We broke the randomisation code when there were 1,601 complete records. We excluded records from respondents who stated they were not participating in the study for the first time (n = 57) or were under 18 years old (n = 16). We included the remaining 1,528 records in the analysis. The participants were evenly distributed across the four comparison groups and the groups were similar with respect to age, sex, education and VAS scores (Table 1). Fifty-three percent were women, compared to 51% in the Norwegian population [25]. Compared to the general population, there were more people 50–59 years old (29% versus 17%) [25] and a higher proportion of participants with university level education (59% versus 23%) [26].

**Table 1:** Participant characteristics.

		Positive framing for 10 years	Negative framing for 10 years	Negative framing per year	No information	Total	Norwegian population*
		*n* = 365	*n* = 369	*n* = 393	*n* = 401	*N* = 1,528	
		%	%	%	%	%	%
**Women**		52.9	52.3	52.9	54.6	53.2	51.0
**Age**							
	18–29	14.8	14.4	11.7	13.5	13.5	19.4
	30–39	15.3	17.9	18.1	15.5	16.7	20.0
	40–49	18.6	17.6	20.1	21.2	19.4	18.3
	50–59	31.0	27.1	29.0	29.7	29.2	17.0
	60–69	15.3	17.9	16.5	16.0	16.4	10.7
	70–79	4.9	4.6	4.3	4.0	4.5	8.6
	over 80	0.0	0.5	0.3	0.2	0.3	6.0
**Education**							
	Elementary	6.6	10.0	8.9	5.7	7.8	31.0
	High school	34.5	31.7	34.1	33.7	33.5	42.7
	University	58.9	58.3	57.0	60.6	58.7	23.3
**Values (on 100-point visual analogue scale)**							
		mean (SD)	mean (SD)	mean (SD)	mean (SD)	mean (SD)	
	CVD	93.2 (12.5)	93.7 (12.2)	94.4 (12.6)	92.6 (12.4)	93.5 (12.4)	
	Side effects	75.0 (25.0)	73.1 (25.8)	74.8 (26.2)	74.6 (25.8)	74.4 (25.7)	
	inconvenience	45.1 (36.8)	45.6 (36.8)	46.2 (37.2)	43.7 (35.9)	45.2 (36.6)	
	RIS	−26.9 (51.9)	−25.0 (52.9)	−26.6 (51.3)	−25.7 (50.8)	−26.1 (51.7)	

*For the Norwegian population, the proportion of women and each age group is based on the population over 17 in 2004 [11]. The proportion of people with different levels of education is based on the highest completed education for people over 16 years old [15].

**Figure 6:**
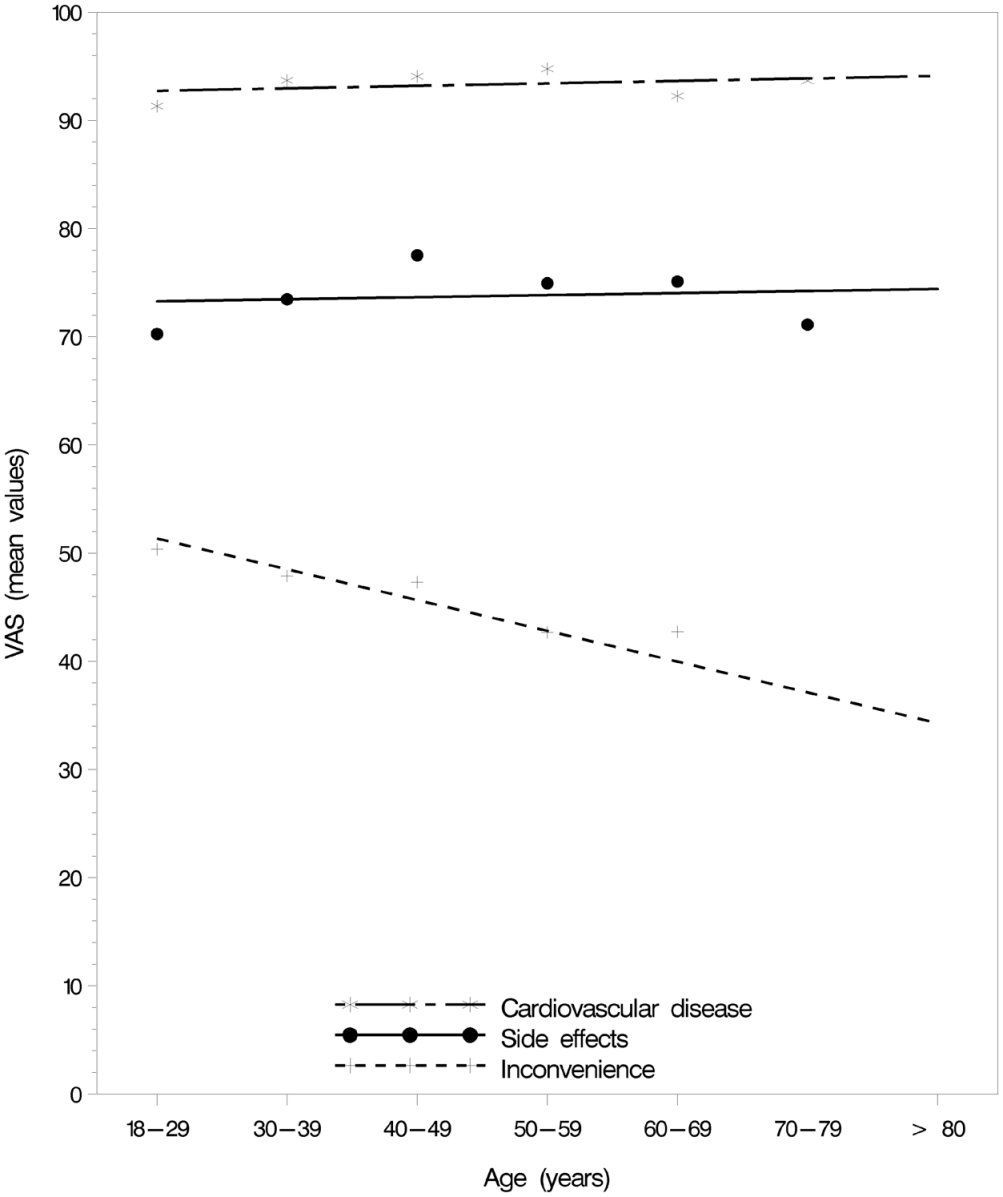
Visual analogue scores versus age. Visual analogue scores (VAS) for the relative importance of avoiding cardiovascular disease, side effects, and the inconvenience of taking antihypertensive medication with lower and upper anchors of “Not important” and “Very important”.

The importance of avoiding CVD and the side-effects of medication did not vary with age. The importance of avoiding the inconveniences of medication was negatively correlated with age (Spearman r = −0.09, *p* = 0.0001) (Figure 6).

There were statistically significant differences (p<0.001) in the proportion of participants who chose to take medication across the four groups (Table 2). The largest proportion was in the no-information group (80.3%) followed by the group shown negatively framed information for 10 years (66.4%) and negatively framed information per year (62.8%). The group that viewed positively framed information had the smallest proportion of participants who chose to take medication (55.9%). Overall, 46.9% chose to take medication after viewing all additional information and all three presentations compared to 66.6% that opted for medication on the first decision.

**Table 2:** Decisions to take antihypertensive medication.

Decision	Positive framing for 10 years	Negative framing for 10 years	Negative framing per year	No information	Total	*P*-value
Responses	*n = *365	*n = *369	*n = *393	*n = *401	*N = *1528	
	% (*n*)	% (*n*)	% (*n*)	% (*n*)	% (*n*)	
**First decision**						
Would take medication	55.9 (204)	66.4 (245)	62.8 (247)	80.3 (322)	66.6 (1018)	<0.001
**Second decision**						
Would take medication	39.7 (145)	45.8 (169)	48.9 (192)	52.6 (211)	46.9 (717)	0.004
**Change from first to second decision**						
From “take” to “not take”	35.8 (73)	36.7 (90)	26.3 (65)	38.5 (124)	34.6 (352)	0.016
From “not take” to “take”	8.7 (14)	11.3 (14)	6.8 (10)	16.5 (13)	10.0 (51)	0.122
Total changes	23.8 (87)	28.2 (104)	19.1 (75)	34.2 (137)	26.4 (403)	

Among those who changed their decision, participants in all four groups were significantly more likely to change from taking to not taking than from not taking to taking medication (p<0.001 for all four groups). Among all those who first answered that they would take medication, 34.6% changed their decision from taking medication to not taking medication. Among those that first answered negatively, only 10.0% changed their decision. There were statistically significant differences in the proportions that switched their decision from taking to not taking medication across the four groups (p<0.001). The largest proportion changed their decision in the no information group (38.5%) and the smallest proportion (26.3%) changed their decision in the group shown negatively framed information per year (Table 2).

### Decisions in Relation to Values

**Figure 7:**
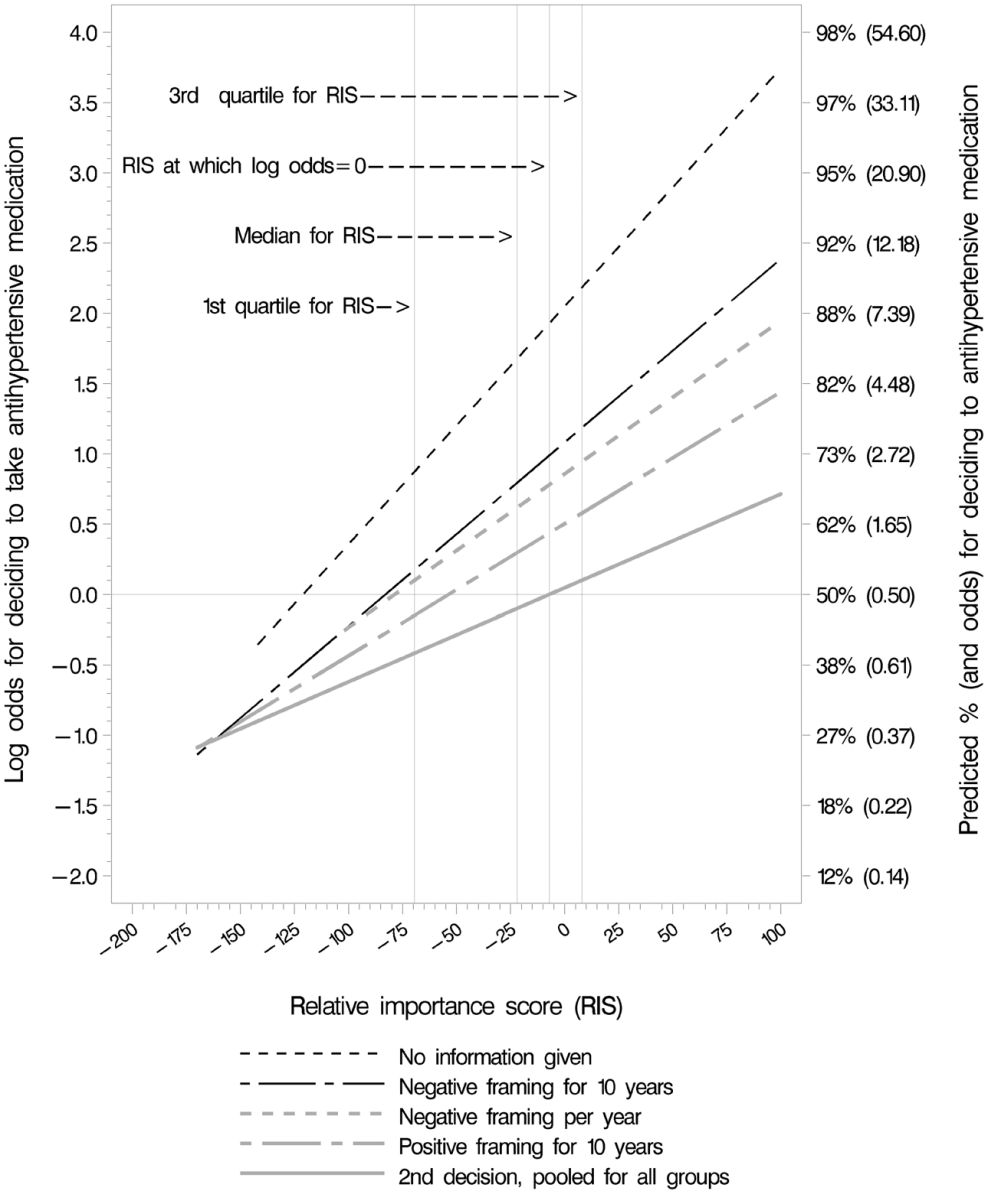
Likelihood of deciding to take medication in relation to RIS. Relative importance score (RIS) values indicate the relative importance to participants of the desirable and undesirable consequences of taking antihypertensive medication. As anticipated, the likelihood of participants deciding to take medicine is greater when the relative importance of the desirable consequences (less risk of CVD) is greater and the relative importance of the downsides of taking medication is less.

There was a clear association between participants' RIS and the decisions they made in all four groups and across groups for the second, more fully informed decision (Figure 7). The likelihood of deciding to take antihypertensive medication increased as RIS scores increased, as expected. The likelihood of deciding to take medication was greatest in the no information group across RIS values (Table 3 and Figure 7). Among the three presentation groups it was greatest in the group shown negatively framed information for 10 years and least in the group shown positively framed information. The interaction between RIS and presentation group was not statistically significant (*p* = 0.2) in the logistic regression model. Therefore the null hypothesis of equal slope of the linear predictors was not rejected. Thus, we report only the ORs for the two pair-wise comparisons that we specified *a priori* (Table 4). The likelihood for deciding to take antihypertensives with positive framing for 10 years versus the likelihood with negative framing for 10 years resulted in a statistically significant odds ratio of 0.63 (*p*<0.004) while the comparison of these likelihoods between negative framing per year and negative framing for 10 years was not significant.

**Table 3:** Likelihoods for deciding to take antihypertensive medication in relation to values (RIS).

Presentation	1^st^ quartile		Median		3rd quartile	
	RIS = −70		RIS = −22		RIS = 8	
	Odds (95% CI)	Predicted % (95% CI)	Odds (95% CI)	Predicted % (95% CI)	Odds (95% CI)	Predicted % (95% CI)
**Positive framing for 10 years**	0.86 (0.66–1.13)	46.3 (39.7–53.0)	1.35 (1.09–1.67)	57.4 (52.0–62.5)	1.78 (1.37–2.33)	64.1 (57.7–69.9)
**Negative framing for 10 years**	1.19 (0.91–1.57)	54.3 (47.5–61.0)	2.21 (1.75–2.80)	68.9 (63.6–73.7)	3.27 (2.42–4.44)	76.6 (70.8–81.6)
**Negative framing per year**	1.10 (0.85–1.43)	52.5 (45.9–58.9)	1.85 (1.49–2.30)	65.0 (59.8–69.7)	2.57 (1.94–3.40)	72.0 (66.0, –77.3)
**No information**	2.39 (1.80–3.18)	70.5 (64.3–76.1)	5.35 (3.95–7.24)	84.3 (79.8–87.9)	8.89 (5.84–13.55)	89.9 (85.4–93.1)
**Second decision (all)**	0.66 (0.58–0.76	39.7 (36.5–43.0)	0.91 (0.82–1.00)	47.5 (45.0–50.1)	1.11 (0.98–1.25)	52.5 (49.5–55.5)

RIS  =  Relative importance score.

Predicted %  =  proportion deciding to take antihypertensive medication based on logistic regression.

**Table 4:** Comparisons of the presentation groups.

Presentation	Odds Ratio (98.3% CI)^a^	*p*-Value
Positive framing for 10 years versus Negative framing for 10 years	0.63 (0.46–0.86)	<0.004
Negative framing per year versus Negative framing for 10 years	0.86 (0.63–1.17)	0.343

a Adjusted overall CI level = 0.95.

Because the interaction term was not statistically significant and the differences in slopes (Figure 7) might be due to chance, we removed the interaction term, thus using a model that assumes the slopes are the same. We compared the odds of a positive decision to take antihypertensives on the first decision for each group to the odds of a positive second decision for the pooled estimate for the other three presentation groups (Table 5). All four groups were significantly more likely to decide to take medication on the first decision.

**Table 5:** Odds ratios for deciding to take antihypertensive medication on the first decision for each group compared to the more fully informed second decision the other three groups (Model without interaction term).

Presentation	Odds ratio (95% CI)	p-value
Positive framing for 10 years	1.33 (1.05–1.70)	0.02
Negative framing for 10 years	2.25 (1.75–2.89)	<0.0001
Negative framing per year	2.02 (1.58–2.57)	<0.0001
No information	5.29 (4.01–6.99)	<0.0001

As only 10% changed their decision from ‘Not taking medication’ to ‘Taking medication’, we used the logistic regression model of whether the respondents changed their decision from ‘Taking medication’ to ‘Not taking medication’ versus ‘Did not change decision’ depending on the RIS, presentation group, and the interaction. Presentation group and RIS were significant variables (*p*<0.0001 and *p* = 0.009), suggesting that the respondents were more likely to change their mind with increasing values of RIS. The interaction was not significant (*p* = 0.8), i.e there was no significant difference between the slope of the linear predictors of the decision switch versus RIS.

## Discussion

In general, as participants' RIS values increased in a direction that would favour taking antihypertensive medication, they were more likely to decide to take medication, regardless of what information they were provided. While the relative importance of CVD and side effects of medication were constant across age groups, the relative importance of the inconvenience of taking medication decreased in relation to the age of the participants.

The majority of the participants (66.6%) chose to take medication in all four groups for the first decision, with statistically significant differences across the groups (from 60% in the group shown positively framed information to 80% in the group shown no information). Only 47% of participants chose to take medication for the second decision, after being more fully informed. The decrease in the proportion of participants choosing to take medication from the first to the second, more fully informed decision for the group shown no information for the first decision suggests that the participants may have assumed that the benefits of antihypertensive medication were greater than they are for a 40 year-old man without other risk factors.

### What Was Already Known and What This Study Adds

Two systematic reviews of the effects of different ways of presenting information to patients included a total of 16 studies investigating the effects of positive and negative framing [2], [3]. Edwards and colleagues found six studies that investigated loss versus gain framing on uptake of screening (i.e., describing the risks or disadvantages of not being screened versus describing the benefits or advantages of being screened) [3]. Uptake of screening was more likely with loss framing compared to gain framing (OR 1.18, 95% CI 1.01 to 1.38 for 4 studies). This is consistent with the prediction that loss-framed messages would be most effective, because of the assumption that detection behaviours are perceived as risky in the short term because of their ability to detect disease [15]. Moxey and colleagues found that framing effects varied with the type of scenario, patient characteristics, scenario manipulations, and study quality [2]. Surgery was more likely to be preferred with positive framing (survival) than negative framing (mortality) (RR 1.51, 95% CI 1.39 to 1.64 for 5 comparisons from 4 studies). Ten studies examined gain versus loss framing for health behaviours, of which three provided data on the proportion undertaking the desired health behaviour. Respondents were more likely to perform the desired behaviour when information was framed as gains compared to loss (RR 1.22, 95%CI 1.04 to 1.43), consistent with what the prospect theory would predict. Overall no significant framing effect was evident for immunization (5 studies). Eleven studies examined positive versus negative framing for medication treatment decisions, but only one study with inconclusive results provided data on the proportion choosing medical treatment. Those with little interest in behaviour at baseline were more likely to be influenced by framing, particularly information framed as gains. Framing effects were less in studies with a lower risk of bias and ones that examined actual decisions.

Our results do not support the prediction of prospect theory that positive framing promotes risk aversive behaviours, such as uptake of preventive behaviours, compared to negative framing for preventive behaviours. In fact, the results support the opposite conclusion. The group shown positively framed information was least likely to decide to take antihypertensive medication. A possible explanation for this is that when risks are small and they are presented as natural frequencies, differences in the number of people with an event (between small numbers) are perceived as larger than differences between the people without an event (between large numbers), even though these differences are the same.

Another explanation is that the participants perceived the prospect of suffering the downsides of taking antihypertensive medication as more risky to their well-being than the risk of suffering from CVD. Other studies have also found that positive framing promotes uptake of preventive behaviours when the undesirable effects are small or not mentioned [e.g. 27], [28] and that the effect of positive compared to negative framing varies with the probability of success [e.g. 29]. Finally, prospect theory was developed to explain decisions where there is one risky choice and one sure thing [10], [30] and it may not apply here.

Nonetheless, the higher odds across all levels of RIS of those shown negatively framed information deciding to take antihypertensive medication compared to those shown positively framed information illustrates a valence framing effect due to violation of the principle of invariance, i.e. people should make the same choices given equivalent descriptions and values [31], but they did not.

Public health advocates might argue that the negatively framed information was “best” since it resulted in the highest proportion of participants deciding to take antihypertensive medication (Table 2). They might, in fact, argue that none of the presentations were satisfactory, since all of them resulted in smaller proportions of participants deciding to take antihypertensive medication compared to the “no information” group. We assumed that the additional information and the cognitive processing required in order to understand it would foster a more systematic use of the information, thereby minimizing the heuristics and biases that might interfere with people making a decision according to their preferences, so that the second, more fully informed decision would best reflect decisions consistent with the participants' values. Although the decisions in the positively framed information group appeared to differ least from the second decision that was made after viewing all three presentations, there was a significantly higher likelihood to decide to take medication on the first decision in all groups (Table 5). Thus none of the three presentations is clearly “best” in terms of helping participants to make decisions that were most likely consistent with their preferences.

Similar proportions of people changed from a decision to take medication to not taking medication in the positively and negatively framed groups over 10 years (36% and 37%), whereas a smaller proportion changed in the group shown negatively framed information per year over 10 years (26%). There were still statistically significant (*p* = 0.004) differences in the proportion of people deciding to take medication on the second decision after all four groups had been shown all three presentations. These findings could be explained by a reluctance of people to change their decision after first making a choice. It is uncertain why participants shown negatively framed information per year would be less likely to change their decision than participants in the other groups. It is possible that the impression of a small difference made by that presentation (7 versus 5–6) elicited a greater feeling of certainty, referred to as the “certainty effect” [14], than the other presentations (70 versus 56, and 930 versus 944) (Figure 2). This would be consistent with prospect theory [14].

### Applicability of the Findings and Implications

The participants were recruited through a popular nationally televised weekly health program and needed to have access to the Internet. TV-recruitment and the randomisation process worked well, generating four comparable groups. There were more than twice as many respondents with university education compared to the Norwegian population (Table 1). It is uncertain that the findings are applicable to populations with less education [2], [23]. The study attracted more men than our previous study using the same recruitment strategy (47% versus 31%), which focused on antibiotic treatment of sore throat, and more participants over 40 years old (70% versus 40%) [24]. Nonetheless, most of the participants were women and only 36% were between 30 and 49 years old. Fifty-four percent of those who started the study did not complete it (Figure 1). We do not have demographic information for those people, although it is likely that many chose not to complete the study because they did not find the scenario relevant.

In this study we chose not to collect additional information about the participants in order not to burden them with questions that were not necessary for the primary analyses, with the hope that this would increase the proportion of people who would complete the study after starting it. Thus, although participants were likely attracted to the study, at least in part, because of a personal interest in antihypertensive treatment, we do not know how salient the scenario was for the participants [23]. It is uncertain to what extent their responses to the hypothetical scenario we used, where they were asked to pretend that they were a 40 year-old man, reflect what they would actually decide [1]–[3], [33], [34]. Context affects the way information is understood and processed [13], so that it is likely that decisions made under hypothetical conditions might differ from real decisions. Nonetheless, responses made under hypothetical conditions may predict real-life behaviour [32].

Although these results have limited relevance to personal communication with an active interaction between a physician and a patient [3], we believe the results are likely to be relevant for electronic and printed patient information and generally applicable to people who are uncertain about whether to take antihypertensive medication for two reasons. Firstly, 55% of participants who were not shown any information for their first decision changed their minds for the second decision, after they were given information, and 45% of all of the participants changed their mind from the first to the second, more fully informed decision (Table 2). This suggests that participants were uncertain and that the information that was provided influenced their decision. Most of those in the “no information” group who changed their mind (91%) changed from a decision to take antihypertensive medication to a decision not to take it. This suggests that most participants (80%) started out assuming that the desirable consequences of taking antihypertensive medication outweighed the undesirable consequences, and many of those participants (39%) changed their mind. This is in contrast with our earlier study where most participants (77%) started out assuming that the desirable consequences of taking antibiotics for sore throat did not outweigh the undesirable consequences.

Secondly, we found that the likelihood of participants deciding to take antihypertensive medication was greater when the relative importance of the desirable consequences (less risk of CVD) was greater and the relative importance of the downsides of taking antihypertensive medication were less (Figure 7). Thus, for people with a relatively low risk of CVD, as was used in our scenario (7% over 10 years), this appears to be a preference-sensitive decision [4], [35] and the hypothetical decisions taken in this study are consistent with what we would expect. These findings support the recommendation that the absolute risk of cardiovascular disease should be used as the basis for discussing with a patient whether drug treatment should be initiated [36].

In this study, negatively framed information appears to have resulted in decisions that were least consistent with decisions that were made by all of the participants after they were more fully informed and had seen all three presentations, but participants shown all three presentations were significantly more likely to have decided to take medication on the first decision. The implication of this is that those preparing and using electronic or printed patient information or decision aids for preference sensitive decisions for people at low risk should be cautious about presenting only negatively framed information. It may be best to present information framed both positively and negatively to help people to reach decisions that are consistent with their own values [20], [37], although presenting both positive and negative frames may lead to information overload [38]. For clinicians, the results suggest that it may be important to take the time to present the benefits of antihypertensive medication in several different ways.

### Conclusions

Our findings suggest that presenting either positive or negative framing alone may result in decisions that are inconsistent with patients' values. Some patients appear more likely to decide to take antihypertensives when their preference is to not. These findings apply to people with a relatively low 10-year risk of CVD and may apply to other low risk situations. In such situations, presenting treatment effects using both gains and losses may help to improve the extent to which patients make choices that are consistent with their values and preferences.

The extent to which these results can be applied to other decisions is not clear. They are most likely to be relevant for Internet-based and printed patient information, and for people at low risk considering interventions that have modest effects and relatively important down sides. Although they are less likely to be relevant in the context of personal communication between doctors and patients, they suggest that it is likely to be important to explore how individual patients perceive and balance reasons for and against taking antihypertensive medication [39].

## Supporting Information

Checklist S1 CONSORT Checklist.
(0.19 MB DOC)

Protocol S1 Trial Protocol.
(0.07 MB DOC)
