# Supplementary material for: The Effect of How Outcomes Are Framed on Decisions about Whether to Take Antihypertensive Medication: A Randomized Trial
Source: PLoS One. 2010 Mar 1;5(3):e9469. doi: 10.1371/journal.pone.0009469 (PMC2830888; doi:10.1371/journal.pone.0009469)
Supplement: Protocol S1 — Trial Protocol. (0.07 MB DOC) [file pone.0009469.s002.doc]

**The effect of how outcomes are framed on decisions about whether to take antihypertensive medication: A randomized trial**

Cheryl Carling, RN, MSc, Research Researcher^1^

Signe Flottorp, MD, PhD, Researcher^1^

Doris Tove Kristoffersen, MSc, Researcher^1^

Andrew David Oxman, MD, Director^1^

Elie Akl, MD, Resident^2^

Phillip J. Deveraux, MD, Research Fellow^3^

Jeph Herrin, PhD, Research Consultant^4^

Tom MacKenzie^5^

Victor Montori, MD, MSc^3,6^

Holger Schünemann, MD, PhD, Assistant Professor^2,3^

1. Informed Choice Research Department

Norwegian Health Services Research Centre, Norway

1. Departments of Medicine and Social and Preventive Medicine

University of Buffalo, USA

1. Department of Clinical Epidemiology and Biostatistics

McMaster University, Canada

1. Flying Butress Associates

Charlottesville, VA, USA

1. Department of Medicine

University of Colorado, USA

1. Knowledge and Encounter Research Unit, Department of Medicine

Mayo Clinic College of Medicine, USA

Address for correspondence:

Informed Choice Research Department

Norwegian Health Services Research Centre

P.O. Box 7004, St. Olavs plass

N-0130 Oslo

Norway

Phone: +47 24 16 32 48

Fax: +47 24 16 30 11

Email: [cheryl.carling@kunnskapssenteret.no](mailto:cheryl.carling@kunnskapssenteret.no)

**BACKGROUND**

Relevant, reliable and accessible information about the effects of interventions is essential for informed choices about healthcare. The manner in which this information is presented affects how it is understood and subsequent decisions.^1,2^ The objective of the Health Information Project: Presentation Online (HIPPO) is to improve communication of information about the effects of healthcare based on randomised trials of alternative ways of presenting this evidence.

The focus of this trial is on how framing of the benefits of medicinal treatment for people with hypertension affects peoples' decisions about whether or not to take antihypertensive medication. The results of this study will help to inform decisions about how best to communicate the effects of treatment to patients and the general public.

Decisions under risk involve choices between acts for which more than one outcome is possible, and the probability of each outcome's occurrence is known.^3^ One of the factors that lead to inconsistent choice of risky options is the framing of outcomes in either positive or negative terms, known as the "framing" effect. The framing postulate of prospect theory suggests that people respond differentially to messages depending on how these messages are framed. Although the information presented in the messages may be factually equivalent, the willingness to incur risk in order to promote a desirable outcome or avoid an undesirable outcome changes depending on how the message is framed ^4^. Health messages can be framed either in terms of potential gains (i.e. advantages or benefits) or in terms of potential losses (i.e. disadvantages or costs).

The nature of medical interventions may be categorized as treatment, diagnosis, and health behaviour, which may be further divided into 3 categories: detection behaviour (screening), prevention behaviour (e.g. immunization), and general health behaviour (e.g. diet, smoking). The main benefit of reducing high blood pressure is the reduction of risk for serious cardio-vascular events, such as coronary infarct and stroke, and thus, taking antihypertensives is a preventive behaviour. According to Prospect theory,^4^ choosing a preventive behaviour would be described as a risk-averse option. Since risk-averse options are preferred when people are considering benefits or gains, when gains are made salient, one would expect the performance of prevention behaviours to be facilitated by gain-framed messages. Conversely, people are risk seeking (i.e. they are willing to take risks) when losses are made salient.

A systematic review separated framing studies into treatment, (e.g. surgery, medication), prevention behaviour (e.g. immunization, skin cancer protection), and general health behaviour (e.g. exercise).^5^  Three of five immunization scenario studies showed a favourization for immunization for positively framed scenarios, one showed favourization with negative framing and the fifth showed no framing effect. Of five studies using preventive behaviour scenarios, three showed a favouring of preventive behaviour with positive or gain frames, while two studies showed no framing effect.

None of these has investigated the relationship between the framing presentation that is used and the extent to which decisions are congruent with individuals' preferences for states that are associated with the decision.

## OBJECTIVES

The objective of this study is to determine which of three ways of framing information

about the effects of antihypertensives on the risk for cardiovascular disease results in decisions that are most congruent with individuals' preferences. (appendix 1: the 3 presentations)

We have two main comparisons that we will test. Is the congruence between respondents’ utilities and their decision about whether to take antihypertensives better

1. for positive framing versus negative framing?
2. for negative framing over ten years versus negative framing for one year?

Another objective is to find out which presentation resulted in decisions that are most consistent with “fully informed” choice, which is modelled on the choice made by all participants after they have seen all presentations and been provided more detailed information.

We will also compare the propensity to take antihypertensives in the framing groups vs a control group that is not given any risk-reduction information.

## METHODS

Study design

The study is a Web-based randomised trial in which participants are randomised to one of three ways of presenting information about the benefits of antihypertensives in lowering risk for cardio-vascular disease, and to one control group that does not receive any information.

Upon logging into the study Website (Appendix), participants will be presented with information about the study and asked to give informed consent to participate. They will view a brief scenario in which they will be asked to imagine that they have hypertension that they have not managed to lower to an acceptable level by making life-style changes. They must decide whether or not to take antihypertensives. Each respondent's utilities for the main benefits and harms will be elicited using a visual analogue scales (VAS) for the importance of each of the following: risk of cardiovascular disease (MI and CVA), side effects of antihypertensives, and the nuisance associated with taking them, e.g. cost, blood tests at the doctor, and taking a tablet every day. They will then be randomly presented with one of the three presentation formats about the effect of antihypertensives in lowering CVD risk and asked whether they would decide to take antihypertensives or not. Thereafter, respondents will be shown all three presentations and provided with more detailed patient information. They will be asked a few questions about themselves and then they will be asked to reconsider their decision.

Inclusion criteria

Only complete responses from persons who are at least 18 years old will be included in the analysis.

Participants will be recruited in Norway through a nationally broadcast weekly health television program, PULS. Viewers will be informed about the study and invited to go to the program's web pages where they can use a link to go to the study website. A reminder will be broadcast on the program one week later.

Allocation

Allocation to not to receive any further information or to one of the three presentations is determined by block-randomisation. The block randomisation is also taking into consideration that each respondent will receive all the three different presentation alternatives in random order at the end of the questionnaire. There are 6 different ordered ways of presenting this information, thus the minimum block size is 24. The randomisation list is generated by use of the random generator provided by http:/www.randomization.com. To ensure blinding, the block size and seed will be kept confidential and will only be available for the system developers during the performance period.

Data collection

Participants enter responses directly by responding to the questions on the HIPPO website. The data generated will be stored anonymously in a database. When the sufficient number of participants has completed the questionnaire, the data will be downloaded, the randomisation code will be broken and the statistical analysis will take place. Only complete answers will be used for the analysis.

Analysis

We have the following variables:

U_S_ = utility of suffering cardio-vascular disease; i.e. 1 – how difficult suffering cardio-vascular disease on a scale from 0 to 1; 1 equals the most difficult

U_A_ = utility of the **adverse effects** of antihypertensives; i.e. 1 – how difficult it would be to have one of the common adverse effects of hypertensives on a scale from 0 to 1; 1 equals the most difficult

U_N_ = utility of the **nuisance** of associated with antihypertensive treatment; i.e 1 – how difficult it would be to get and take antihypertensives on a scale from 0 to 1; 1 equals the most difficult

The disutility is given by:

S = (1-U_A_) + (1-U_n_) - (1-U_S_) = VAS_A_ + VAS_N_ – VAS_S_

The main objective of the study will be to identify which of the three presentations results in decisions that are most consistent with what individuals would be expected to decide based on their disutility. When evaluating the decision whether to take antihypertensives or not, the probability for this should have a fairly high value for people who have more concern about having cardio-vascular disease, whereas more concern about adverse effects and the nuisance associated with antihypertensive treatment should be reflected in a low probability. The data will be analysed using a binary logistic regression model to identify which of the summary statistics presentations result in the above. If G is used to represent the presentation group to which a participant is randomised and D represents the decision to take antihypertensives or not, then the relationship between D, G and S, can be modelled as follows:

(Eq 1)          logit(D) = β_0_ + β_1g_G_g_ + β_2_S + β_3g_G_g_*S; g=1,2,3

If there is a relationship between the decision and the presentation group and the disutility, these will be revealed as significant variables in the model.

When considering the regression lines for each presentation group as a function of the disutility, one might think that the presentation group providing the steepest slope should be identified as the group giving the best congruence between the respondents’ disutility and their treatment choice. This however, will not be the most reliable result for a group if it has a very high proportion of individuals deciding to take antihypertensives independent of the disutility score and vice versa if a high proportion of individuals decide not to take antihypertensives.

At the end of the questionnaire the respondents are presented with more information and all of the three different presentation wordings and once more asked to decide whether to take antihypertensives or not. This answer will be regarded as the correct one for establishing the relationship between the decision and the disutility of the respondents across all randomisation groups. Thus, the slopes of the logit(D) per presentation group will be compared to the slope for the second decision. Further, the logit (D) for each of the presentation groups will be compared at the disutility value for which the logit of the second decision is equal to zero, i.e. the odd ratio of the decision ‘Take’ versus ‘Not take’ antihypertensives is equal to one meaning that the proportion of ‘Take’ and ‘Not take’ are equal. The presentation group which provides a logit(D) closest to the logit of the second decision with respect to slope of the regression line and location will be identified to be the best to provide decisions that are most consistent with what individuals would be expected to decide based on their disutility. In addition, we will look at the distance between the linear predictors for two disutility values at each side of the ‘zero value’, located some distance from the ‘zero value’ and a proper number of respondent should have values in this area. These two point should not be values establish from extreme values from the VAS scales. One of the points should then indicate a relatively strong aversion to the down sides of taking antihypertensives and, at the same time, excluded potentially extreme disutility values and the opposite for the second point.

Another measure will be to calculate and compare the area under the ROC curve when considering the second decision as the golden standard. The presentation group providing the largest volume is the best one.

Sample size

It is difficult to provide a proper sample size estimate since we have no previous information about people’s own values and their decision for the use of antihypertensives. We have performed two similar trials; one concerning the use of statins and one concerning the use of penicillin to treat sore throat. However, the information is presented in different ways in all these trials and the disutility is of course different, so the power calculation will provide just a rough estimate. By using the estimate of one of the largest difference in slope (estimate=1.22 and standard error=0.42, test observator χ^2^ =8.4) for two of the presentations in the trial evaluating the use of penicillin, and calculating backward, we achieved a power slightly above 80% when including about 350 patients in each group. If a difference between two of the slopes is as big as this or bigger, this will be detected by including a total of 1400 respondents. If the difference is smaller more respondents are needed to claim the difference statistically significant at the same power.

Additional analysis

If sufficient data are available, age, gender and level of education will be included in the model to test whether the inclusion of any of these provides a better fit. If significant, differences between the groups will be estimated.

## Ethics

Participants are informed on the consent screen that they can leave the study at any time, and are given the option of choosing to have any data that they might have entered deleted.

Confidentiality of data entered by participants is ensured by not collecting any information that would make it possible to identify participants. If participants fill out forms to indicate an interest in participating in future studies, their email addresses will not be stored in the same database as their responses nor will these databases be linked in any way.

Both the Norwegian Data Protection Agency and the Medical Ethics Board have cleared the study.

## REFERENCES

1. McGettigan P, Sly K, O’Connell D, Hill S, Henry D. The effects of information framing on the practices of physicians. J Gen Intern Med. 1999; 14:633-42.
2. Herrin J, Schünemann H, Oxman AD, Vist G, Olsen K. Presentation of empirical evidence about health (Cochrane Review). In: The Cochrane Library. Oxford: Update Software. Under revision.
3. Baird, BF. Introduction to decision analysis. North Scituatem, MA: Duxbury Press, 1978.
4. Kahneman D, Tversky A. Prospect theory: An analysis of decision under risk. Econometrica, 1979, 47, 263-291.
5. Moxey A, O'Connell D, McGettigan P, Henry D. Describing treatment effects to patients: How they are expressed makes a difference. J Gen Intern Med 2003;18:948-959.
